# Moving forward the Effects of Gene–Diet Interactions on Human Health

**DOI:** 10.3390/nu14183782

**Published:** 2022-09-14

**Authors:** Valentini Konstantinidou, Sarela Garcia-Santamarina

**Affiliations:** 1 MEDOLIALI S.L. (DNANUTRICOACH®), Calle Almogavers 165, 08018 Barcelona, Spain; 2 Faculty of Health Sciences, Universitat Oberta de Catalunya (Open University of Catalonia, UOC), 08018 Barcelona, Spain; 3 Instituto de Tecnologia Quimica e Biologica (ITQB NOVA), Universidade Nova de Lisboa, 2780-157 Oeiras, Portugal

Back in 2010, when we first published data on the in vivo nutrigenomic effects of virgin olive oil polyphenols within the frame of the Mediterranean diet [1], the field of nutritional genomics, nutrigenetics, and nutrigenomics was at its birth. Today, this field is generating increasing attention, and consumers seem to be increasingly interested in understanding how general nutritional recommendations may be shaped by their unique genetic fingerprints.

Research is growing at a fast pace, acknowledging that gene–diet interactions play an enormous role in human health and are typically only partially assessed. Healthcare providers, on the other hand, receive scarce knowledge of these interactions, thus jeopardizing the application of precision nutrition [2,3]. At the same time, market initiatives have been developing to fulfill the market’s increasing interest in prevention through nutrition in order to optimize people’s health.

This Special Issue on the effects of gene–diet interactions in human health includes six original research articles showing data that will bring readers closer to the state-of-the-art developments in the field of nutritional genomics. From human intervention studies in Spain and Latin America to large-scale genome-wide association studies (GWAS), such as the UK Biobank, and the study of molecular mechanisms in Drosophila melanogaster, this issue includes the latest advances driving our understanding of gene–diet interactions.

A genetic correlation and two-sample mendelian randomization study was performed by Xu et al. [4] assessing the association between 143 dietary habits and osteoporosis. The authors presented seven candidate dietary habits that showed genetic associations with osteoporosis. Hjorth et al. [5] analyzed gene expression changes in liver cells to assess the postprandial effects of salmon fishmeal and whey, and serum metabolic markers, in a randomized trial of five healthy male participants. The authors did not detect any differentially regulated genes after either of the protein source or postprandial time.

Granado-Serrano et al. [6] studied dietary fiber supplementation in a three-arm intervention study with 63 Spanish volunteers aged 37–68 years old. Fiber supplementation and/or fiber-rich diets have shown controversial results regarding their cholesterol-lowering effects [7]. The authors of this work studied the changes in fecal microbiota and blood lipids profile after treatment with three different fiber supplements. Their results led them to conclude that lack of response to fiber treatment could be mediated by the inability of non-responders to maintain a stable diversity of short-chain fatty acid producing bacteria.

Rivera-Iñiqguez et al. [8] studied how a nutrigenetic intervention impacted emotion, rewarding behaviors, and self-efficacy in an exploratory pilot study in Mexico, which is a topic that has not yet been thoroughly assessed. They included twenty-eight individuals during a 6-month intervention. The authors concluded that when dietary advice is culturally acceptable and genetically compatible, it can reduce negative emotions and unhealthy eating behavior while increasing self-efficacy. These results open a wide new field of recommendations that psychologists and therapists could use to treat people with unhealthy and emotional eating behaviors.

A high polygenic risk score for Type 2 diabetes mellitus was used by Lopez-Portillo et al. [9] to assess the association between fasting glucose and sugar-sweetened beverages intake in a Chilean population. Their weighted genetic risk score was based on 16 SNPs related to T2DM in 2828 non-diabetic participants. They found that the association between intake of sugar-sweetened beverages and fasting glucose levels in this population was modified by their genetic susceptibility to develop T2DM.

Du et al. [10] used Drosophila melanogaster to study how prolonged salt consumption is linked to the disruption of the biological process of the circadian rhythm. Instead, they found that a high-salt diet impaired neuronal plasticity in the axonal projections of circadian pacemaker neurons. Their findings were tested in human studies and revealed the exact mechanisms by which excess salt intake impairs physiological and neurological behaviors beyond high blood pressure.

Gene–diet interactions, once fully incorporated into clinical practice, hold great promise as a precise tool to improve quality of life, health span, and reduce healthcare costs. This special issue added a piece of knowledge in that direction with more precise data and analysis.

## References

[1] Konstantinidou V., Covas M.I., Muñoz-Aguayo D., Khymenets O., de la Torre R., Saez G., Tormos Mdel C., Toledo E., Marti A., Ruiz-Gutiérrez V. (2010). In vivo nutrigenomic effects of virgin olive oil polyphenols within the frame of the Mediterranean diet: A randomized controlled trial. FASEB J..

[2] Joffe Y., Herholdt H. (2020). What Will It Take to Build an Expert Group of Nutrigenomic Practitioners?. Lifestyle Genom..

[3] Kaufman-Shriqui V., Salem H., Boaz M., Birk R. (2020). Knowledge and Attitudes towards Nutrigenetics: Findings from the 2018 Unified Forces Preventive Nutrition Conference (UFPN). Nutrients.

[4] Xu J., Li S., Zeng Y., Si H., Wu Y., Zhang S., Shen B. (2022). Assessing the Association between Important Dietary Habits and Osteoporosis: A Genetic Correlation and Two-Sample Mendelian Randomization Study. Nutrients.

[5] Hjorth M., Galigniana N.M., Ween O., Ulven S.M., Holven K.B., Dalen K.T., Sæther T. (2022). Postprandial Effects of Salmon Fishmeal and Whey on Metabolic Markers in Serum and Gene Expression in Liver Cells. Nutrients.

[6] Granado-Serrano A.B., Martín-Garí M., Sánchez V., Riart Solans M., Lafarga Giribets A., Berdún R., Vilaprinyó E., Portero-Otín M., Serrano J.C.E. (2022). Colonic Microbiota Profile Characterization of the Responsiveness to Dietary Fibre Treatment in Hypercholesterolemia. Nutrients.

[7] Bozzetto L., Costabile G., Della Pepa G., Ciciola P., Vetrani C., Vitale M., Rivellese A.A., Annuzzi G. (2018). Dietary Fibre as a Unifying Remedy for the Whole Spectrum of Obesity-Associated Cardiovascular Risk. Nutrients.

[8] Rivera-Iñiguez I., Panduro A., Villaseñor-Bayardo S.J., Sepulveda-Villegas M., Ojeda-Granados C., Roman S. (2022). Influence of a Nutrigenetic Intervention on Self-Efficacy, Emotions, and Rewarding Behaviors in Unhealthy Eating among Mexicans: An Exploratory Pilot Study. Nutrients.

[9] López-Portillo M.L., Huidobro A., Tobar-Calfucoy E., Yáñez C., Retamales-Ortega R., Garrido-Tapia M., Acevedo J., Paredes F., Cid-Ossandon V., Ferreccio C. (2022). The Association between Fasting Glucose and Sugar Sweetened Beverages Intake Is Greater in Latin Americans with a High Polygenic Risk Score for Type 2 Diabetes Mellitus. Nutrients.

[10] Du X., Yu L., Ling S., Xie J., Chen W. (2021). High-Salt Diet Impairs the Neurons Plasticity and the Neurotransmitters-Related Biological Processes. Nutrients.

